# Medical Opinion and Movement

**Published:** 1911-02-11

**Authors:** 


### Medical Opinion and Movement.

### Pancreatic Lesions in Diabetes.

At a meeting of the Medical Society of Vienna, Dr. Weichselbaum reported on the researches he has carried out on the development of the islands of Langerhans in the pancreas, and on his observation in 183 autopsies on patients suffering from diabetes. The islands of Langerhans have the same origin as the rest of the parenchyma of the glands, but do not develop in glandular parenchyma. They are regenerated, not at the expense of the parenchyma, but by proliferation of the insular cells or by transformation of the small excretory ducts. In general the pancreas of the diabetic shows no macroscopic changes. Sometimes there is an atrophy and sometimes a fatty infiltration. On microscopic examination, one generally finds a diminution in the number and volume of the islands. The microscopic change is of four kinds. In subjects under forty it consists in a hydropic degeneration which is followed by atrophy and then disappearance of the insular cells. In patients over fifty the change consists in a sclerosis of the islands and is generally accompanied by a sclerosis of the pancreatic vessels. A third type is shown by hyaline degeneration of the insular cells. It occurs at an advanced age, and is also accompanied by vascular sclerosis. In the fourth type there are hæmorrhages in the islands often accompanied by regeneration or hypertrophy of the islands. According to the author such lesions are not found in any other condition except diabetes. The failure of many observers to discover such changes in diabetic cases is explained by Dr. Weichselbaum on the ground that in many cases there is only the hydropic degeneration, which may easily escape observation.

### Pituitary Extract for Uterine Inertia.

Dr. Dale has already shown that pituitary extract has the property of directly stimulating contractions of the unstriated muscle of certain organs, and Drs. Frankl-Hochwart and Fröhlich have demonstrated that it is able to excite contractions of the bladder and uterus, independently of any rise in blood-pressure. Acting on these suggestions, Dr. J. Hofbauer, of Königsberg, has tried the effect of subcutaneous injections of pituitary extract in twelve cases of uterine inertia. He injected 0.60 to 1.30 gramme. About five minutes after the injection the uterine contractions, which were previously feeble and at long intervals, become strong and at short but regular intervals, without however any sign of tetanisation of the uterine muscle, such as is liable to follow other means employed for the same purpose. The injection may be repeated. Although the author used the extract undiluted there was no sign of intoxication or local irritation. As pituitary extract also acts on the bladder, Dr. Hofbauer has used it also for this purpose after delivery of the child in cases in which natural micturition did not take place. He finds that in such cases injection of the extract generally produces a normal micturition in a few minutes.

### Hydrogen Peroxide for Cystitis.

The utility of hydrogen peroxide solution in the treatment of all manner of suppurative conditions is already well known, but its use for lavage of the bladder in conditions of cystitis has not hitherto been suggested. One might suppose that the evolution of gas which takes place when the hydrogen peroxide comes in contact with a suppurative surface would be liable to produce undesirable consequences. This does not appear, however, to be the case. Dr. A. Weith, of Lucerne, has tried the remedy in a single case and is much impressed with its efficacy. The patient was a man aged eighty-four, suffering from enlarged prostate and a purulent cystitis. Treatment by lavage with boric acid solution, and occasionally with a weak solution of silver nitrate, failed to effect much improvement in the course of about three weeks. Dr. Weith then had recourse to treatment with hydrogen peroxide. He commenced cautiously and first washed the bladder out with 200 grammes of hydrogen peroxide of only two volumes concentration. The result was entirely satisfactory. Bubbles of gas escaped freely but caused no pain to the patient, only a feeling of warmth. The condition was so much improved that no further lavage was carried out for three days. After two applications the strength of the hydrogen peroxide was increased to three volumes, and at the end of another three weeks the patient had completely recovered. Although the experience of one case is insufficient for any definite conclusions, the result certainly justifies a more extended trial of hydrogen peroxide lavage in cases of cystitis, as its efficacy in other suppurative processes is already so well established.

### Hydrogen Peroxide for Hyperchlorhydria.

Reference has already been made in these columns to the treatment of dyspepsia with hyperacidity by the administration of hydrogen peroxide, when the idea was first suggested by Dr. Petri and successful results were also published by Dr. Goodman. Since then the treatment has been pursued by other Continental physicians, and further satisfactory reports are to hand. Dr. Girardi, of Turin, has tried the method in fourteen cases, and is persuaded of its efficacy. He gives 15 to 20 c.c. of hydrogen peroxide in 150 c.c. of water about half an hour after meals. Dr Poly, of Wurtzburg, has similarly treated forty patients suffering from hyperchlorhydria without complications, such as gastric ulcer or gastritis. His method is, however, somewhat different. Every other day after a test meal he gives 300 c.c. of 0.5 per cent. solution of hydrogen peroxide and then examines the gastric contents, and according to the result determines whether the treatment should be continued. He finds that in the majority of cases three to five administrations of the hydrogen peroxide are sufficient to cause a disappearance of the trouble. In cases in which the hyperchlorhydria is more serious, amounting from

0·24 to 0·36 per cent. free hydrochloric acid, the dose must be repeated ten or more times, but he finds that it is just in such cases that the treatment is especially efficacious. So far as can be judged at present, the treatment appears to be permanent in its effects. Dr. Poly, examining some of his patients several weeks after treatment, found the gastric secretion still quite normal.

### Pregnancy with Fibroids.

The tendency of recent obstetric innovation has been distinctly towards early operative interference whenever fibroids are discovered complicating pregnancy. A more conservative note is struck in the *American Journal of Obstetrics* by Dr. Lobenstine, who at the end of a careful paper sums up thus: The great majority of the cases that do not abort early proceed through pregnancy, labour, and the puerperium with few or no symptoms; therefore, operative interference is but rarely indicated during pregnancy, and is, as a rule, meddlesome midwifery. This statement he makes cautiously, for he knows it will be received with some degree of criticism. The operation of myomectomy during pregnancy is just now very popular, but he believes it to be very rarely indicated, for the results of it are none too good. It is, of course, admittedly necessary to do myomectomy sometimes during pregnancy, and also hysterectomy, but as a rule conservatism should be the watchword. The proportion of cases in which no apparent dystocia occurs, but normal labour instead, is extraordinarily high; still, in delivering, great care must be taken to avoid all possible injury to the tumour. If during the puerperium symptoms of gangrene in a myoma should develop, then early operation is obligatory. In cases of abortion due to fibroids, there are cases so difficult to handle *per vias naturales* and so dangerous owing to hæmorrhage, that laparotomy is the operation of choice for them.

### A New View of Schlatter's Disease.

The attention which was drawn by the writings of Schlatter in Germany and Osgood in America about the same date (1903) to cases of painful enlargement of the tubercle of the tibia in adolescents has resulted in the recognition of the fact that the condition is not at all uncommon. Both these observers held that muscular violence is responsible for the complaint, which is commonest in athletic boys, by tearing off the epiphysis of the tubercle, or a portion of it. Mr. R. C. Elmslie controverts this view, with regard to some, at least, of the cases, in the *British Journal of Children's Diseases*. According to recent authorities the separate centre for the tubercle is only rarely present; but whether this or the older view is correct, at any rate about fourteen years of age the tubercle consists of a tongue-like process extending down from the anterior part of the upper epopysis of the tibia and separated from the shaft by a layer of cartilage. This fact is well illustrated by some *x*-ray pictures reproduced. That injury may be not the only factor in the ætiology of Schlatter's disease is suggested by two bilateral cases which Mr. Elmslie reports. In other cases no history of injury can be obtained, and in yet others the condition supervenes on occupations requiring kneeling. He believes that the lesion, instead of being a partial traumatic separation, is a chronic inflammatory process, leading to enlargement of the cartilaginous tubercle and irregularities in its ossification. *X*-ray evidence is adduced in support of the condition, which requires to be further studied by skiagraphy of adolescents.

### Serum Disease.

A hitherto unpublished case of death due to anti-diphtheritic serum furnishes Dr. Raymond Wallace with a text for an important discourse on this subject in the *Medical Record*. He points out that up to the past year the leading authorities have not recognised the seriousness of some cases of serum disease, though they are now beginning to modify the views expressed in text-books only two or three years ago. The specific case which he describes is that of a neurotic man, aged twenty-nine, who received 1,000 units of antitoxin from a syringe, the other half of whose contents was injected into his wife. No air was injected, and the wife exhibited no untoward effects whatever. Five minutes after the injection he began to vomit, came out in large confluent urticarial wheals, manifested acute dyspnœa with general œdema, and in thirty minutes was dead. No autopsy was permitted; but it is stated that there was no paralysis, nor any sign suggesting a cerebral vascular lesion. Dr. Wallace's conclusions are as follows: There is occasional danger of sudden death in sensitised individuals or those subject to bronchial asthma or to respiratory distress in other forms. Individuals peculiarly susceptible to the odour of horses or stables, and those subject to asthmatic attacks, hay-fever, and bronchitis, should have horse serum administered only upon most urgent cause, and then with due prophylaxis. Insufficiency or inadequacy of the suprarenal glands may account in the asthmatic type for the peculiar reaction. The administration hypodermically of aqueous extract of the suprarenals previous to the use of serum in this class of case is desirable in order to rehabilitate and control the vasomotor system, and may prove of great prophylactic value, as well as useful in the heroic treatment of the acute attacks of serum disease. The desquamation following serum disease should not be confounded with that of scarlet fever.

### Tabes Dorsalis and Cystitis.

There are few of the complications of locomotor ataxia which the practitioner learns to look out for with such watchfulness and such concern as that of cystitis. Probably an ascending urinary infection is the most fertile cause of the actual final illness in sufferers from this disease; there are few tabetics who escape it altogether. In a paper abstracted for the *Medical Record*, Dr. Barney presents his experience of bladder infection complicating tabes.

Trabeculation is found in more than 80 per cent. of cases, and may occur before any symptom or other sign of vesical disturbance. In typical cases of tabetic bladders it is found in the fundus and side walls only; it is more delicate and regular than that seen with mechanical obstruction. It is probable that it is due to a primary detrusor hypertrophy. Residual urine is found in more than 70 per cent. of cases, often before any symptoms begin. In over 50 per cent. of all cases the urine is infected. Cystoscopy is generally a painless proceeding, a fact which is of some diagnostic value. Urinary disturbances, variable in character, are observed in over 90 per cent. of all patients with tabes.

### Poisoning by Eucalyptus Oil.

Oil of eucalyptus and chloroform have a recognised value in the treatment of ankylostomiasis, but their use is at times attended with somewhat alarming phenomena, and several cases have been reported of late in which symptoms of poisoning occurred. G. G. Jolly, I.M.S., reports another example in the *Indian Medical Gazette*. The patient —a Hazara—was admitted to hospital for anæmia and dyspepsia. His blood showed a well-marked eosinophilia, and ova of ankylostomum duodenale in considerable numbers were subsequently found in the stools. An aperient of magnesium sulphate was given at night, and on the following morning a mixture containing oil of eucalyptus, 30 minims; chroloform, 45 minims; and castor oil, 10 drachms, one-half being given at 7 A.M., the second at 7.30 A.M. At 9.30 A.M. the patient complained of weakness and giddiness, and at 10 A.M. was in a state of profound collapse, great langour, giddiness, difficulty of respiration, pain all over the body, and especially in the stomach, head, and urethra. Respiration was shallow and laboured, pulse weak, irregular and of low pressure, pupils rather dilated, and the conjunctival reflex diminished. A mustard plaster was applied over the heart and a hypodermic injection of strychnine administered. At 11 A.M. the patient was no better, and the extremities were cold. Atropine sulphate $\frac{1}{200}$gr. was given subcutaneously, and hot bottles applied to the feet. Shortly afterwards he passed a copious motion. An enema was given, which was followed by two other motions. The patient rapidly got better and soon recovered.

### Spinal Injections of Mercury.

A new method of introducing mercurial salts into the body was described at a recent meeting of the Paris Academy of Medicine by Drs. Lévy-Bing and G. Lévy. These investigators have recently had to deal with a number of cases of syphilis of the spinal cord, and it struck them that a more rapid effect than usual might be caused on the affected tissues by injecting mercurial salts directly into the spinal canal. The results of this local treatment have been encouraging, but are not at present such as to make the method one to be adopted in all cases of the kind. In the first place the injections are accompanied by a severe degree of pain, not only at the moment, but for several hours afterwards. Œdema of the lower limbs and even paralysis have followed the treatment, not to mention vertigo, fever, and urinary complications. In any case it is well for a patient to stay in bed for forty-eight hours after the injection. On the whole the observers think that the results obtained are not so much better than those which follow the more usual forms of treatment as to justify such risks. In general paralysis and advanced tabes the results are nil, and the most that can be expected is a slight improvement when intensive treatment is employed in the very early stages of the latter disease—that is to say, when diagnosis is still doubtful. On the other hand, however, the method is very satisfactory in cases of syphilitic meningitis and myelitis.

### The Pain-signal in Gastroptosis.

Under the above title Dr. G. Leven, in *La Clinique*, describes a method of determining clinically the lower boundary of the stomach. The procedure is based on the fact that pain due to pressure on a hyperæsthetic solar plexus, and making its appearance near the middle line of the body at a point between the xyphoid process of the sternum and the umbilicus, is relieved and at times abolished from the moment the finger touches and lifts up the lower border of the stomach. No complicated manœuvre is required to bring about this result. The patient should be made to stand up against a wall in front of the doctor, who should be seated on a high stool. The point of maximum pain should then be made out by deep palpation. Two fingers of the patient, or, better still, of an assistant, should be placed on this point and made to elicit pain by pressure. The physician should then endeavour to lift up the abdominal contents by deep pressure made with both his thumbs placed tip to tip, against the abdominal wall, commencing near the pubic-symphysis, and gradually approaching the umbilicus. As soon as the patient announces the diminution or disappearance of the pain the observer will know that the lower limit of the stomach has been reached. The counter-proof can be elicited by beginning above and making pressure in a downward direction. Pain will persist as long as the thumbs are pressing above the lower level of the stomach. The exactitude of these observations has been proved by radioscopic examination, so that this method of examination can be made use of when the $x$-rays cannot be obtained, though, of course, it is only of value in cases where the stomach is diseased or dilated, for solar hyperæsthesia is necessary for the pain-signal to be elicited. In this way the exact point can be made out at which to place an abdominal support. The author has shown by radioscopic investigations carried out with Dr. G. Barret that the majority of such supports, instead of elevating, depress the stomach.

